# Food insecurity and ageing: rising risks among older Asian Americans

**DOI:** 10.1017/S1368980026101992

**Published:** 2026-02-11

**Authors:** Jeff Clyde G. Corpuz

**Affiliations:** Department of Theology and Religious Education, College of Liberal Arts, De La Salle University, 2401 Taft Avenue, Manila 1004, Philippines

Dear Editor,

The recent study by Nhan *et al.* on food insecurity among older Asian Americans sheds light on a growing but often overlooked concern in public health nutrition^(1)^. Using data from the California Health Interview Survey (2011–2018), the authors demonstrated that food insecurity is not evenly distributed across Asian American subgroups and that older adults bear a disproportionate burden. Among older Vietnamese adults, food insecurity prevalence reached 26 %, while older Chinese adults also reported high levels at 17 %. Across all subgroups examined, older adults experienced significant increases in food insecurity between 2011–2014 and 2015–2018, in contrast to declining rates among younger adults^(1)^.

The disparity between age groups is especially concerning. For example, older Chinese adults were four times more likely to experience food insecurity compared with younger Chinese adults. Between 2011 and 2018, prevalence increased by 45 % among older Vietnamese and 25 % among older Chinese. These patterns suggest that aging itself, combined with fixed incomes, chronic illnesses and physical limitations, places older adults at greater risk of nutritional hardship than younger groups^(2)^.

Several social and structural risk factors compound this vulnerability. Being foreign-born, lacking citizenship, speaking a language other than English at home and not having health insurance were all associated with higher rates of food insecurity. Participation in Supplemental Nutrition Assistance Program (SNAP) was limited, and those enrolled were still more likely to experience food insecurity, reflecting both underutilisation of available benefits and the inadequacy of current support. Cultural stigma, language barriers and limited access to appropriate foods may further restrict the effectiveness of assistance programmes for older Asian Americans^(1)^.

Recent findings deepen the significance of these results. Wu and colleagues^(3)^ reported that during the COVID-19 pandemic, food insecurity decreased among adults who received enhanced SNAP benefits but did not decline among non-SNAP participants. Importantly, racial and ethnic disparities persisted, highlighting both the protective role and the limitations of SNAP. Older Asian Americans, who are often under-enrolled due to stigma and lack of awareness, may not have fully benefited from these pandemic-related expansions^(3)^.

The nutritional dimension of food insecurity also deserves attention. Aljahdali found that food insecurity was linked to higher consumption of ultra-processed foods, with the direction of this relationship differing by race^(2)^. For older adults, including Asian Americans, the combination of limited food access and reliance on processed options may worsen chronic disease outcomes and accelerate functional decline. These insights extend the conversation from food quantity to food quality, reframing food insecurity as a barrier not only to sufficiency but to healthy aging^(2)^.

Future research should clarify how food insecurity interacts with diet quality and health outcomes among older Asian Americans. Longitudinal studies can track how shifts in food access affect chronic conditions and daily functioning, while evaluations of culturally adapted food programmes may help identify effective interventions. Policy responses should focus on improving SNAP outreach, ensuring access to culturally appropriate and nutritious foods, and embedding food security assessments into routine geriatric care.

Food insecurity among older Asian Americans has often been rendered invisible by assumptions of economic stability within this population. The evidence provided by Nhan et al.,^(1)^ together with recent findings on SNAP and dietary patterns, makes clear that hidden hunger in later life is both real and increasing. Addressing it will require better data, culturally sensitive interventions and policy measures that place nutrition security at the centre of strategies for healthy aging.
